# Construction Notes

**Published:** 1894-09-15

**Authors:** 


### CONSTRUCTION NOTES.

**Blackburn Fever Hospital.**

This hospital, just opened, is situated at Longshaw, in the borough of Blackburn. The site of 10½ acres is enclosed by a boundary wall. A stream runs through this, affording a good outlet for the subsoil drainage. On the left of the entrance gates there is a porter's lodge, an office, kitchen, scullery, two bed-rooms, bath, lavatory, &c. The out bathing-place is opposite the lodge, and to the right of the entrance. There are two waiting-rooms, bath, lavatories, &c. It is intended this should be used principally as the final disinfecting and discharging place for patients. A patient, before being discharged, will be admitted into one of the waiting-rooms, will bathe, and then receive his disinfected clothing. The administrative block, situated to the left of the main entrance, is three storeys high, and contains accommodation for the medical officer, matron, and nurses, with 16 bed-rooms, baths, lavatories, &c. Hydrants are placed on the landings of all floors of this block. A detached portion of the administrative block, one storey high, contains scullery, dairy, pantry, larder, &c., and is placed in such a position as to afford easy access to the various wards in which provisions, &c., are required. The pavilions, two in number, consist of long, one-storeyed buildings divided into two main wards by the nurses' room and scullery. All angles between floors, walls, and ceilings have been carefully rounded off. The scarlet fever pavilion lies to the right of the administrative block, and contains the following wards: Men's ward, affording accommodation for 10 beds; a women and children's ward, with accommodation for 14 beds. A private ward to each of these contains one bed. The two main wards are separated by the nurses' room. There is also an isolation reception ward and small ventilating engine house at the entrance of this pavilion. Lavatories, bathrooms, moveable baths, sprays, &c., are provided in both the men's and women's wards. A verandah has been erected on the west side. The wards are heated by Manchester grates placed in the centre of the floors. The isolation block is situated immediately to the left of the administrative block, and contains three single wards, nurses' room, with baths, sinks, &c. An interesting feature of the hospital is the ventilation of the scarlet fever pavilion. This has been carried out by the Sturtevant Company, and combines heating with ventilation. The cubic air-space allowed per bed is 2,000 feet. The disinfectory, the stabling, and the mortuary blocks face the west of the site. The disinfector was supplied by Messrs. Goddard, Massay, and Warner. There are 16 beds provided in the administrative block for the staff. The plans of the hospital were prepared and carried out by the borough engineer, Mr. J. B. McCallum, member of the Inst.C.E.; the contractor for the masonry being J. Whittaker. The total cost, including land and furniture, has been about £21,000.

**Wood Green Cottage Hospital.**

The foundation-stone of this cottage hospital has recently been laid by Mr. Passmore Edwards. The scheme appears to have originated with the local committee of the Hospital Saturday Fund. Mr. Passmore Edwards generously offered £2,000 towards the building. The new structure is to be in the style of an old English cottage, the walls being of yellow stock bricks, the upper part being rough cast, and the gables tiled. There will be two large wards, each containing four beds, and two single wards for isolation purposes, making ten beds in all, an operating room, matron's room, kitchen, scullery, and necessary offices, also bed-rooms for matron, nurses, &c., and a mortuary in the rear of the main building. The total cost is estimated at £2,500, including the site, towards which the Ecclesiastical Commissioners will contribute £300. The building is being carried out from plans prepared by Mr. Charles Bell, F.R.I.B.A., and by Mr. Charles Wall, contractor.

